# Environmental judgment in early childhood and its relationship with the understanding of the concept of living beings

**DOI:** 10.1186/2193-1801-2-87

**Published:** 2013-03-07

**Authors:** Jose Domingo Villarroel

**Affiliations:** Escuela Universitaria de Magisterio de Bilbao, University of The Basque Country, Sarriena s/n. 48940-Leioa, Bizkaia, Spain

**Keywords:** Early environmental education, Moral reasoning, Emotion, Animacy

## Abstract

The evidence collected concerning the *biocentric* judgment that young children express when evaluating human actions on the environment leads some scholars to suggest that an essential understanding of the notion of living beings should appear earlier than previously believed.

This research project aims to study that assumption. To this end, young children’s choice when they are put in situation of having to compare and choose the most negative option between environmentally harmful actions and the breaking of social conventions are examined. Afterwards, the results are categorized in relation to those obtained from the study of children’s grasp of the distinction between living beings and inanimate entities.

The data is analysed according to the individuals’ age and overall, it suggests a lack of relationship between environmental judgment and the understanding of the concept of living beings. The final results are discussed in keeping with recent research in the field of moral development that underscores the role that unconscious emotional processing plays in the individual’s normative judgment.

## Introduction

Nowadays childhood environmental education is attracting increasing attention and is being considered as a significant focus of research activity (Davis 2009, 2010; Kopnina 2011; Kossack & Bogner 2012; Lee & Kang, 2012; Onura et al. 2011; Williams et al. 2012). The increase in global environmental concern and the subsequent growth in the number of environmental awareness initiatives have been pointed out as crucial factors in this interest aimed towards children’s consciousness of the environment (Hussar & Horvath, 2011).

In this respect, a significant line of research has attempted to examine the relationship between biological knowledge and environmental awareness. Regarding this topic, a considerable amount of research has been undertaken across different educational levels, for instance, in primary education (Mutisya & Barker, 2011), in secondary education (Rioux, 2011) and also, among undergraduates (Arora & Agarwal, 2011; He et al. 2011) and adults (Robelia & Murphyb 2011).

Regarding the earliest educational levels, a wide area of research has been conducted to examine young children’s grasp of the basic biological concepts and, especially, how the notion of living being evolves during childhood (see for instance: Inagaki & Hatano, 2008; Margett & Witherington, 2011; Leddon et al. 2011; Lee & Kang, 2012 and Osborne & Freyberg, 1985). Moreover, from another separate perspective, profuse research activity has been undertaken in the study of young children’s judgment on the environment (Ergazaki & Andriotou, 2010; Hussar & Horvath, 2011; Severson & Kahn, 2010).

Nevertheless, as far as our knowledge extends, no research has been carried out regarding the relationship between young children’s environmental consciousness and their conception of living kinds. This is the case, even though some research claims that young children would not be able to form judgments related to harmful actions against the environment that have been recorded, without a basic understanding of the distinction between living beings and inanimate entities (Severson & Kahn, 2010).

In view of this, the scope of this essay is the emergence of the understanding of the concept of living things among children between 4 and 6 years old, their environmental judgement and the reflection on the role that individual’s normative judgment may play in the emergence of the concept of animacy.

The following provides a concise review on the current state of research on the subject of how young children build the concept of alive. After that, an overview of key issues regarding the study of young children’s environmental judgment is presented. This initial chapter will finish by introducing the objectives of this research.

### The emergence of the concept of living beings

The living being concept is a remarkable scientific notion that raises the possibility of building an integrated understanding of biological knowledge. Unsurprisingly, it has been a focus of reflection from both a theoretical perspective (El-Hani, 2008) and the field science education (Caravita & Falchetti, 2005; Schroeder et al. 2010; Yorek et al. 2009).

Moreover, the understanding of how human beings learn to classify some entities as living beings, as opposed to those which are referred to as inanimate objects, is a recurrent research issue in the field of developmental psychology (Woodward et al. 2001). Likewise, the study of this cognitive ability has proved to be very influential in other areas of research, such as those connected to human cognitive impairment (Zaitchik & Solomon, 2008) or to the origin of the human cognitive system (Tsutsumi et al. 2012).

Focusing on the study of how the comprehension of the concept of living being is developed during childhood, much of the recent research has been carried out challenging the Piagetian perspective of animacy (Piaget, 1929). According to this paradigm, children’s limitations when handling non evident cause-and-effect relationships that underlie many biological phenomena and, also, the ontological egocentrism that characterizes children during the preoperational stage (Kesselring & M&üller, 2011) allow very little room for the consideration that young children’s concept of animacy might have some degree of internal coherence, consistency and predictive value (Solomon & Zaitchik, 2012).

In contrast to this account, in recent times an extensive and also diverse research endeavour has been undertaken seeking to overcome the developmental limitations posed by the Piagetan view. This line of research is based upon the assumption that conceptual development is conditioned, but not limited, on the one side, by an innate cognitive nucleus, which is common to all human beings (Spelke & Kinzler, 2007), and, on the other side, by social and cultural experiences (Scheinholtz et al. 2010).

 (Keil, Accordingly, the interaction between these two factors would boost intuitive explanatory frameworks, also the so-called *naïve theories*^2010^), that consist of a “systems of interrelated concepts that generate predictions and explanations in particular domains of experience” (Murphy, 1993). This primordial body of beliefs concerning particular phenomena might not coincide with scientific perspective but it serves the crucial purpose of relating particular events to wider generalizations and it involves causal explanations and abstract entities.

In this respect, one of the most salient standpoints is related to the so-called vitalistic-causality conception. This view states that at some point between the age of 4 and 8 children give up the behavioral understanding of living things, which appears linked to the existence of volitional activity (Carey, 1985). At this moment, they start to form explanations in which the internal structure of organisms and the importance of nutrients, water and air stand out (Inagaki & Hatano, 2008; Slaughter & Lyons, 2003). A characteristic feature of this type of thinking is that children rely on the existence of some kind of *energy* or *life force* which is inherent in the essential substances to support life and, moreover, that they attribute some kind of intentionality to our organs in order to sustain life (Lindeman & Saher, 2007).

Furthermore, an alternative point of view underscores the fact that the ability to categorize objects as animate and inanimate entities emerges spontaneously very early during development, even during the first months of life (Molina, Van de Walle, Condry & Spelke, 2004). Additionally, it has also been reported that human beings share this cognitive ability with other nonhuman primates (Tsutsumi et al., 2012).

This evidence suggests an essentialist standpoint that states the existence of an inborn cognitive structure which is supposed to be predisposed in human beings from the earliest stages of the development to identify living things and to interact with them.

Presumably, this early ability to pay special attention to living things, and especially to human beings, might have an undeniable adaptive value and is considered as a consequence of the fact that even babies can spot some crucial features related to movement patterns and physical characteristics linked to animate entities (Sanefuji, Wada, Yamamoto, Shizawa, Matsuzaki, Mohri, Ozone & Taniike, 2011).

Finally, another line of research has focused on the study of teleological explanations that children show in their comprehension of biological phenomena and their understanding of living things. These explanations are characterized by the fact that individuals interpret natural phenomena based on assumptions regarding objectives, designs or purposes for which different agents (living beings, biological or geological events, organs, etcetera) have been created (Kelemen et al. 2005). It is worth noting that invoking teleological explanations to account for natural phenomena is not an exclusive feature of children’s thinking. Much to the contrary, adults also frequently use this kind of thinking when attempting to make sense of a broad range of biological and geological phenomena (González & Meinardi 2011; Kelemen & Rosset, 2009).

On the whole, the presented areas of research address the study of the process of constructing the notion of living being from the perspective of the individual’s cognition. In this context, how individuals give sense to the notion of animate entities and what kind of explanatory theories they use to distinguish living beings from inanimate objects are the main areas of study.

However, what remains a subject of debate within literature is the way in which children progress through the different conceptions of living things and what kind of social practices have the potential to boost children in this progression until reaching a coherent significance with scientific perspective (Leddon et al., 2011; Schroeder et al., 2010).

### Early environment judgment in childhood

Moving on to the examination of the main lines of research concerning the study of young children’s environmental judgment, most of the available research on this topic is related to the issues of, firstly, whether children hold moral reasoning when it comes to judging harmful actions against nature and, secondly, whether the judgement they produce is linked to a human-centred framework or whether, on the contrary, they are apt to employ nature-centric arguments.

Regarding the first point, the most significant research starts out from a cognitive-developmental approach and more specifically, from Turiel’s social-domain theory (Turiel 1983; Smetana, 2006). According to this theoretical perspective, moral reasoning is related to the development and coordination of the three different but decisive domains of knowledge regarding normative reasoning: moral domain (concerning the physical or psychological harm that can be caused to others), social-conventional domain (linked to social norms, rules or traditions) and psychological domain (related to personal choices such as leisure time, clothing or friends).

Previous cognitive-developmental approaches in the field of moral psychology stated that an individual’s normative reasoning progresses from a preconventional initial moral standpoint in which acts are considered right or wrong on the basis of expected punishment, to the highest moral stages in which rules are justified by abstract and universal principles (Kohlberg, 1969; 1981). In contrast to this standpoint, Turiel’s social-domain theory regards that knowledge in each of the domains (moral, social-conventional and personal) determines the subject’s normative reasoning and additionally, that the development of these domains run parallel from early childhood.

Regarding the study of young children’s environmental judgment in concordance with Turiel’s theory, recent research accounts for the fact that children consider environmentally harmful behaviour worse than social-conventional transgressions but, likewise, they consider actions against a human beings’ psychological or physical welfare (that is, moral transgressions) as the most objectionable. Consequently, this line of research strongly suggests a different normative domain for environmental judgment, which would be separated from moral, socio-conventional and psychological domains (Hussar & Horvath, 2011).

Moreover, complementary data regarding young children’s environmental judgment refers to the finding that young children are able to use *biocentric reasoning* (namely, judgments linked to the idea that the environment is worthy of some kind of moral status -Schmidt, 2011-) when it comes to judging environmental transgressions (Ergazaki & Andriotou, 2010; Hussar & Horvath, 2011; Severson & Kahn, 2010).

In this regard, in a significant study regarding 4 and 5 year old children’s justifications against forest fire, Ergazaki and Andriotou (2010) emphasize the idea that children’s *biocentric* (Ergazaki & Andriotou, judgment concerning the environment needs to have any relationship with their understanding of biological concepts. More specifically, this study report a significant number of young children who sustain their opinions on the basis of “flora-centric” criteria such as: *“plants can grow like us … we must let them grow”*^2010^, p. 194). These authors conclude that the use of this kind of reasoning by young children suggest that they have to handle some basic knowledge about the distinction between living and nonliving things.

These thoughts are certainly in line with recent research which confirms that young children demonstrate not only a basic biological framework for differentiating living and nonliving kinds, but also a more sophisticated understanding of plants as living beings than previously thought (Margett & Witherington, 2011).

Furthermore, it is worth noting that these ideas contrast with the well-documented fact concerning the limited knowledge that young children display about plant life and, also, the difficulties that they find when it comes to attributing life status to plants (Gatt et al. 2007; Leddon et al. 2009; Opfer & Siegler, 2004). It has even been suggested that there is a progressive development of the animacy concept according to which the concept of life is granted firstly to human, then to animals and after that, to plants (Yorek et al. 2009).

Summing up, some studies report that even young children award a particular moral status to living creatures in the environment, including plants. This fact leads some scholars to suggest that children possess some basic knowledge of the notion of living being around which they can structure their *biocentric* justifications. However a significant number of studies state that the understanding of the notion of living beings, especially when it comes to considering those of which have a stationary nature, is thought to be beyond the comprehension of young children.

### Objectives of the research

According to the framework presented in this introductory chapter, this research aims to provide additional data regarding the link between young children’s environmental judgement and the understanding of the notion of living being.

With this in mind, the objectives proposed in this study are as follows. Firstly, the research will analyse young children’s understanding of the concept of living being by means of testing their ability to distinguish living beings from inanimate objects.

Moreover, the study will attempt to determine whether young children judge harmful actions against nature more severely than the breaking of social conventions and, consequently, whether they regard undesirable behaviour against the environment to be more of an equivalent to moral transgressions.

Subsequently, the aforementioned data concerning both the comprehension of the concept of living being and environmental judgment will be examined in relation to the age of the children.

Finally the study reflects on the relationship between the understanding of the concept of living being that the children of the sample show and the pattern of choice that they express concerning the alternative between actions against the environment and violations of social conventions.

## Methods

### Characteristics of the sample

As regards the sample of this study, it is comprised of 118 children (52 boys and 66 girls). Of these, 35 (29.7%) were in the first level of preschool education (4–5 year old); 40 (33.9%) were in the final stage of preschool education (5–6 year old) and 43 (36.4%) were enrolled in the first course of primary education (6–7 year old).

All the children in the sample analysed were in the appropriate academic level according to their age.

The subjects of the sample attended three state schools located in three different towns with more than 3,500 inhabitants in the region of Uribe-Coast, in the Basque province of Biscay, Spain (Beck, 2006).

A singular cultural feature of the Basque community is the co-existence of two official languages: Spanish (which it shares with the other provinces in Spain) and Basque, which is at present spoken by around 900,000 people in the Basque Autonomous Community, the Regional Community of Navarre (also in Spain) and south western France (Cenoz, 1998). Bearing in mind this characteristic, the meeting with children were mainly conducted in Basque, which is the academic language in the three schools involved in this study.

All these schools were visited in the first quarter of 2012. The research protocol was agreed and approved by the principal of each of the schools involved in this study and the parents of the children involved in the research were informed by the direction board of each school concerning the purpose and method of the study and also regarding the procedure for expressing the wish not to take part in the study. Nobody among the families whose children were to participate in the research project refused to cooperate with the study. Furthermore, no video or audio recording was made in order to protect the anonymity of participants in the research.

### Procedure and description of tasks

The research methodology used to undertake this study is based on individual interviews. However, initially a group session was carried out with the objective of introducing the children to the activity proposed and to give them the opportunity to become familiar with the researcher.

This preparatory session was conducted in the presence of the teachers and in the participant’s usual classroom. It usually started with a short dialogue with the children in which the researcher introduced the objective of the task. In this regard, the participants were informed that they would be asked to make a drawing to give to a puppet that the researcher brought with him. In addition, the researcher told the children he would ask them a few questions about some pictures that the puppet had also brought. It is worth noting that the children’s drawings were not the objective of this research. However drawing and colouring is very familiar to young children and an activity which they are fond of (Karniol, 2011). Therefore, this activity was used to start the individual meetings as a means of creating a friendly and comfortable atmosphere that encourages young children to partake in the whole of the activity proposed.

Similar preparatory sessions in which puppets are used as stimulus to support children’s involvement in a research activity regarding young children’s understanding of scientific notions have been successfully used in other studies (i.e., Villarroel et al. 2011).

In order to avoid influence from other classmates, the individual meetings took place outside the classroom but always as close as possible to the classroom in order to make them feel comfortable.

As previously mentioned, the interviews were stated with the proposal of the undertaking of a drawing. After that, insofar as the researcher observed that the child began to feel comfortable; he introduced the *environmental judgement test*, firstly, and, then, the *living/non living distinction test*, then. The characteristics and procedures followed regarding these tests are presented below.

### The environmental judgment test

The design of this task is based on the Hussar and Horvath (2011). By means of individual interviews and by showing pictures in which different examples of rule breaking appeared, the aforementioned authors examined young children’s judgments regarding rule transgressions in four different domains: moral, socio-conventional, personal and environmental in accordance with Turiel’s social-domain theory (Turiel 1983; Smetana, 2006).

The *environmental judgment test* developed to conduct this research is founded upon a similar methodology. To this end, six pictures concerning example of rule breaking that could be easily familiar to the children were carefully chosen from educational books published for young kids. Among these pictures, two presented distinctive moral transgressions, another two displayed socio-conventional rule breaking and, finally, two more illustrated damage to the environment and, more specifically, to plant life. Table 1 breaks down a detailed description of the pictures used, the kind of rule transgression that each picture represents and the source of the images.

**Table 1:** **Detailed information regarding the pictures used to conduct the*environmental judgment test***

	The description of the situation	The type of transgression	The source of the picture
Picture 1	A child picks up another child by the collar while violently threatening to strike.	Moral transgression	Thomas & Harker (2000)
Picture 2	A child takes a sweater from another’s schoolbag without permission. The owner has her back to the offender and is not aware of what is happening.	Moral transgression	Thomas & Harker (2000)
Picture 3	A girl is picking her nose.	Social-conventional transgression	Aliki (1990)
Picture 4	A boy is eating soup so fast that it flies out of the dish, dirtying the table.	Social-conventional transgression	Aliki (1990)
Picture 5	A flower is about to be stepped on by a cartoon character.	Transgression concerning the environment	Gomboli (1997)
Picture 6	A heart is being carved on a tree trunk by means of a knife by a cartoon character.	Transgression concerning the environment	Gomboli (1997)

Unlike the Hussar and Horvath (2011) that also used pictures to examine the domain of personal choices, in this case no images were used connected to this normative area. As Knight (2010) highlights, the distinction between “moral” and the “conventional” normative domains turns out to be the most salient milestone in the development of children’s normative sense. In agreement with that idea, this research aims to delve into the analysis as to whether children’s judgments regarding damage to plant life are related to the moral domain or, on the contrary, children are more prone to consider these kind of environmental damage as a socio-conventional matter.

The procedure designed to conduct the *environmental judgment test* consisted of the following two parts:

Firstly, the abovementioned images were introduced by the researcher one by one and in a random order. While showing these pictures, the interviewer described the scene to the child, just to ensure that child understood the example of rule breaking displayed. It is worth noting that the researcher did not add any moral or normative consideration when describing the scenes in the pictures. Afterwards, the interviewer asked the child whether in her or his opinion, the situation displayed in each picture was *right* or *wrong*. The way to pose the question alternated between *“right”* or *“wrong”* and *vice versa*, with each child interviewed.

In the second part of the *environmental judgment test*, the same previously shown illustrations were used but, in this case, the children were asked to look at two pictures at a time. After that, they were encouraged to compare the two situations displayed in the pictures and subsequently, to choose which of the two situations was the worst. Table 2 presents the pairs of comparisons proposed.

**Table 2:** **Description of the comparison used in the second part of the*environmental judgment test***

The description of the situations to compare	The type of transgressions compared
Picture 1 ***versus*** Picture 3: *Violently threatening a colleague****versus****nose picking*.	*Moral****versus****Conventional transgression*
Picture 4 *versus* Picture 2: *Eating soup too fast****versus****Taking another’s sweater w/o permission.*	*Conventional****versus****Moral transgression*
Picture 3 ***versus*** Picture 5: *Nose picking****versus****Stepping on a flower.*	*Conventional****versus****Environmental transgression*
Picture 6 ***versus*** Picture 4: *Carving a tree trunk****versus****Eating soup too fast.*	*Environmental****versus****Conventional transgression*

*For the full description of the Pictures, see Table*1*.*

The experimenter took note as to whether children considered the situations displayed in each picture as right or wrong. Furthermore, regarding the second part of the test, which of the two situations presented was considered the most negative option was registered.

### The living/non-living distinction test

The final part of the interview was related to the study of the ideas that children in the sample have on *living beings* by means of a categorization task. Following the methodology used by Leddon et al. (2009) and in connection with methods used in previous studies (Osborne and Freyberg, 1985), children were presented with some photographs depicting living and non living entities and they were encouraged to classify each entity as living or non living. Table 3 accounts for the entities depicted in the photographs used.

**Table 3:** **List of the entities that appeared on the photographs used to conduct the*Living/non-living distinction test***

The images used		Category
A tree	Two flowers	Plant
A dog	A bird	Animal
A motor	A car	Vehicle
Some clouds	The sun	Atmospheric agents

It is worth taking into consideration the importance of the words used in the living *versus* non living distinction test. In this respect, Leddon et al. (2009) underscore that children seem to perform better if the question *“Is X a living thing?”* is used, instead of *“Is this X alive?”*.

In accordance with this, the translation of Leddon’s proposal into either Basque (“X biziduna da?”) or Spanish (“Es X un ser vivo?”) were used to carry out the *living/non living distinction test* among the children of the sample studied.

The experimenter recorded the children’s answers for each photograph, taking note of the children’s successes and errors when it comes to considering whether the entity that appeared in each of the pictures was a living or non living thing.

### Data analysis

The quantitative analysis was carried out via a Chi-square test to examine the relationship between nominal variables. Moreover, the analysis of the deviations of the observed frequencies from a theoretical random distribution was carried out by a Binomial test in the case of the study of two categories and by Chi-square test when more categories were involved.

The level of significance used in the study was 0.05 and statistical work was done using the SPSS version 19 software.

## Results

Concerning the presentation of the results of the study, first of all the data obtained from the *environmental judgment test* will be introduced and then, the figures related to the analysis of the *living/non-living distinction test*.

In both cases, the presentation of the results will start with the data linked to the examination of the whole of the sample, followed by the analysis of the differences among individuals belonging to different age groups.

In this regard, three different age groups will be considered: 4–5 year old children, born in 2007 and in their penultimate year of preschool level; 5–6 year old children, born in 2006 and attending their last year of preschool level and, finally, 6–7 year old children, born in 2005 in their 1^st^ year of primary education.

### The environmental judgment test

There was a general agreement among the children of the sample studied regarding the incorrectness of the six types of behaviour in question (see Table 1 for more details about the conduct considered in the meetings with the children of the sample).

Thus, nobody considered that *“nose picking”* was appropriate conduct and only 5 children expressed that “*carving a tree trunk with a knife”* was appropriate. Regarding the rest of the actions (“*violently threatening a colleague”*, “*taking a sweater without permission from another’s schoolbag”*, “*eating too fast”*, “*stepping on a flower”*) in all these cases 117 of the 118 children interviewed associated these types of conduct as undesirable behaviour.

The following provides the analysis of the responses given by the children of the sample when they were put in the situation of having to compare and choose the most negative option between the two alternatives (see Table 2 for more details concerning the comparisons proposed in the interviews).

With reference to, the contrast between moral transgressions and social-conventional violations, the following three categories were considered:

- The *moral choice* category referred to children who judged the two moral transgressions presented during the interview more severely than the breaking of the two social conventions. Accordingly, the subjects belonging to this category always indicated that “*violently threatening a colleague”* and “*taking a sweater without permission from another’s schoolbag”* were more objectionable than “*eating too fast”* and *“nose picking”*.
- The alternative option is from the *social conventional choice* category and it is linked to the belief that the breaking of the two social conventions shown in the pictures was more serious than the two moral transgressions. Therefore, the responses were classified in this category when children had no doubt that “*eating too fast”* and *“nose picking”* were more severe than “*violently threatening a colleague”* and “*taking a sweater without permission from another’s schoolbag”*.
- The indeterminate category gathers those cases in which in only one of the comparisons shown, the child considered that the moral transgression was worse than the breaking of social convention and, consequently, the alternative was the choice related to the other two images compared.

**Figure 1:**
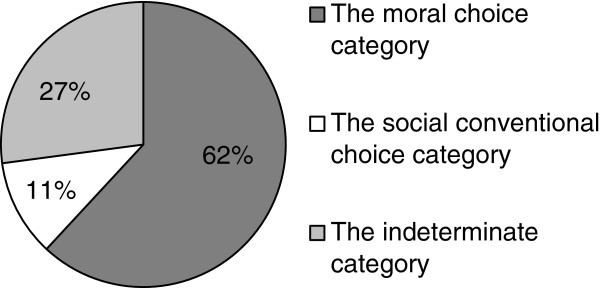
**The relative frequency of the three categories of responses registered regarding the study of the comparison between moral transgressions and the breaking of social conventions among the children of the sampled (N = 118).**

Figure 1 displays the frequencies found in the sample concerning the three aforementioned categories.

It is worth highlighting that the above mentioned pattern of frequencies does not fit into what might be expected in the case of the subjects’ random choices (Chi-Square =100,2 [2]; p < 0.001).

Furthermore, Table 4 breaks down the results of the analysis of how these three categories vary among individuals belonging to different age groups.

**Table 4:** **Relative frequency of the three categories considered related to the comparison between moral transgressions and the breaking of conventions in the age groups studied**

	4-5 (N = 35)	5-6 (N = 40)	6-7 (N = 43)
The *moral choice* category	45.7	72.5	65.1
The *conventional choice* category	14.3	15	4.7
The *indeterminate* category	40	12.5	30.2

Regarding the data provided in Table 4, it is worth noting that when it comes to considering whether children might have reached their choices at random, significant differences were found with respect to a random distribution of the frequencies in all of the age groups (Chi-Square =18.2 [2]; p < 0.001 in the 4–5 age group; Chi-Square =48.8 [2]; p < 0.001 in the 5–6 age group and Chi-Square =45.8 [2]; p < 0.001 in the 6–7 age group).

However, it should be noted that no differences were found in the variation of the frequencies among the three age groups.

In conclusion, the children of the sample did not arbitrarily express their preferences regarding the comparisons between moral transgressions and the breaking of conventions. Additionally, children of different age groups show a similar pattern of choice.

When having to choose the most negative option between environmentally harmful actions and the socio-conventional rule breaking, the responses were classified according to the following three categories:

- The *environmental choice* category when individuals always judged more seriously “*carving a tree trunk with a knife”* and “*stepping on a flower”* (the two environmentally harmful conduct displayed in the pictures) than “*eating too fast”* and *“nose picking”* (the two social conventions illustrated).
- When children expressed the contrary belief; that is, that the two socio-conventional rule breakings were worse than the two types of environmentally harmful behaviour, their answers were classified in the *social conventional choice* category.
- Once again, the remaining cases were categorized as *indeterminate*.

**Figure 2:**
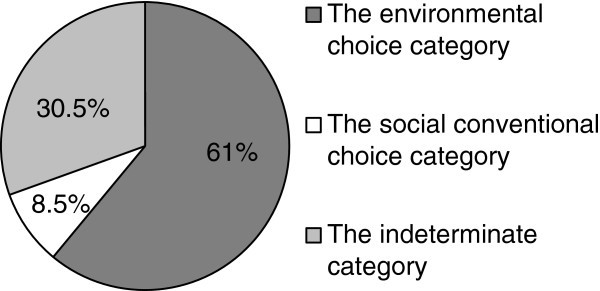
**The relative frequency of the three categories of responses registered concerning the study of the comparison between environmentally harmful actions and the breaking of social conventions among the children of the sample (N = 118).**

Figure 2 displays the frequencies found in the sample concerning the three aforementioned categories.

As was previously the case, there appears to be no basis for believing that children randomly made the choice between the variants *environmentally harmful behaviour* and *social-conventional transgression* (Chi-Square =103.3[2]; p < 0.001).

Moreover, Table 5 presents the results of the analysis of the frequencies in the abovementioned categories among the individuals belonging to the age groups studied in this research.

**Table 5:** **The frequency of preference regarding the comparison between actions against the environment and the breaking of conventions in the age groups studied**

	4-5 (N = 35)	5-6 (N = 40)	6-7 (N = 43)
The *environmental choice* category	60	60	62.8
The *social conventional choice* category	5.7	12.5	7
The *indeterminate* category	34.3	27.5	30.2

Neither the pattern of responses expressed by the 4–5 year old children (Chi-Square =32.1[2]; p < 0.001), nor by the 5–6 year old children (Chi-Square =30.9[2]; p < 0.001) and that which corresponds to the oldest children (Chi-Square =40.9[2]; p < 0.001) are consistent with a model of the random distribution of frequencies.

Furthermore, no differences have been detected concerning the analysis of the variation of the frequencies observed in Table 5 among the age groups.

Summarizing this section, the data presented is not consistent with the belief that children might have answered randomly between environmentally harmful conduct and the breaking of conventions. Moreover, the pattern of responses of the sample studied seems to be irrespective of the children’s age.

### The living/non-living distinction test

The following is the results of the study regarding the children’s ability to correctly categorize living beings and inanimate objects (see Table 3 for details about the images used to carry out this test).

The criteria employed to classify the children’s answers were as follows: a child needed to successfully classify the two entities shown in each category as living or, when appropriate, a non-living thing in order to regard the classification as correct. If not, even when he or she failed once, the researcher registered that the child in question had not achieved the full understanding of the living/non-living distinction in the corresponding category.

For instance, if a child held the idea that the dog illustrated was a living being and expressed the same idea concerning the bird, the researcher considered that the child had successfully performed in the *Animal* category. On the contrary, if a child regarded the flower as a living being but was not able to express the same about the tree, the researcher concluded that the child had performed badly in the *Plant* category. According to this criterion, there was a 25% chance of randomly guessing right.

**Figure 3:**
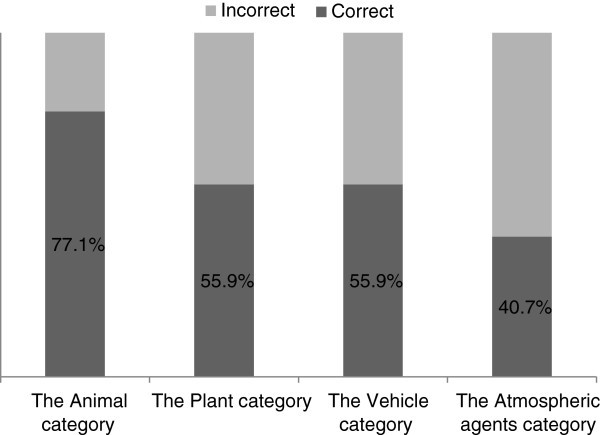
**The relative frequency of the three categories of responses registered regarding the study of the understanding of living being notion among the children of the sampled (N = 118).**

Figure 3 accounts for the figures found in the whole of the sample examined regarding the accuracy of the responses registered.

The above mentioned pattern of frequencies differs significantly from a randomly produced model (the *Animal* category, p < 0.01, binomial test); the *Plant* category p < 0.01, binomial test; the *Vehicle* category, p < 0.001, binomial test and the *Atmospheric agents* category p < 0.001, binomial test).

Moving on to the examination of the differences that individuals from different age groups show concerning the living/non-living distinction test, nobody in the 4–5 and 5–6 year old groups (the preschool educational levels) were able to correctly classify all the entities presented in the six photographs. However, 39.5% of the 6–7 year old children managed to rightly classify all the entities in the photos.

Table 6 breaks down the frequency of the correct classifications (according to the criteria presented at the beginning of this section) achieved by children belonging to different age groups in each of the categories of the entities.

**Table 6:** **The relative frequencies (%) of the correct classifications in the*living/non-living distinction test*related to age groups**

	Age groups		
	4-5 (N = 35)	5-6 (N = 40)	6-7 (N = 43)
The *Animal* category	51.4*	75*	100*
The *Plant* category	34.3	50*	79.1*
The *Vehicle* category	42.9*	37.5	86*
The *Atmospheric agents* category	37.1	22.5	60.5*

* Significant differences with respect to a random choice.

As it is possible that children responded randomly to the *living/non-living distinction test*, the abovementioned frequencies of correct classifications were compared to what would be expected from a random distribution of frequencies (that is, a 25% chance of success).

The binomial test indicates that the classifications given by 4–5 old children regarding the *living/non-living distinction test* were substantially different from what might be expected in a random choice only in the *Animal* (p < 0.01, binomial test) and *Vehicle* (p < 0.05, binomial test) categories. Regarding the *Vehicle* category it is worth noting that in this age group, children more often expressed the idea that the car and the motor were “living beings” than the correct alternative.

Furthermore, the 5–6 year children’s correct classifications had not been achieved randomly in the *Animal* (p < 0.01, binomial test) and *Plant* categories (p < 0.01, binomial test). Finally, with respect to the 6–7 year old children’s classifications, in this case, neither of the categories fits into a pattern of random choice (*Animal* category p < 0.001, binomial test; the *Plant* category p < 0.001, binomial test; the *Vehicle* category p < 0.001, binomial test and the *Atmospheric agents* category, p < 0.001, binomial test).

Concerning the study of the variation of frequencies observed among the three age groups, significant differences have been found in the *Animal* category (Chi-Square =25.9 [2]; p < 0.001); the *Plant* category (Chi-Square =16.5 [2]; p < 0.001); the *Vehicle* category (Chi-Square =23.8 [2]; p < 0.001) and the *Atmospheric agents* category (Chi-Square =12.6 [2]; p < 0.01).

In short, there is enough evidence to state that in the sample studied the children belonging to different age groups performed differently in *the living/non-living distinction test*. Thus, the data collected illustrates a better understanding of the concept of living being expressed by the oldest children, since their responses are not only significantly more correct in comparison to those provided by the youngest children’s but they do not answer arbitrarily in the four categories of the entities considered. Furthermore, the subjects that correctly classified the whole of the entities shown in the test belong exclusively to the 6–7 year old age group.

At the other end of the scale, the youngest children demonstrate a noteworthy lack of comprehension regarding the notion of living beings. They seem to answer randomly when considering whether the sun, plants and clouds are or not living beings. This point suggests that these individuals still do not grasp the criteria to decide which of the aforementioned entities are or not living beings. More interestingly, the 4–5 year old children mostly attributed a “living being” status to vehicles which, according to the data presented, cannot be explained as a consequence of a pattern of random choice.

Finally, the data linked to the results provided by the children belonging to the 5–6 year old age-group is consistent with the assumption that these subjects express an intermediate understanding of the issue of living being in comparison to the other two age groups. Thus, they did not randomly answer on the topic of whether the animals and plants are or not living being and, also, they largely agreed that these entities are in fact living beings. However, when it comes to considering whether the sun, vehicles and clouds are living beings or not, children responded randomly but, in contrast to the youngest children’s answers, they did not regard the entities in the Vehicle category as living beings, only as one might expect according to a pattern of random choice.

## Discussion and conclusion

The discussion of the results will start by addressing the conclusions of the data provided by *the environmental judgment test* and, separately, by *the living/non-living distinction test*. Thereafter, an integrated vision of information collected by both tests will be offered. The closing section of the chapter will cover the implications for future research.

The research resulting “from the social domain theory” (Smetana, 2006 ; Turiel, 1983, 2002) has led to the conclusion that moral judgment (that is, the evaluative beliefs linked to justice, others’ wellbeing and rights) is formed early in their development. Accordingly, it is thought that even young children are aware that conventions are contingent on social systems and more susceptible to be changed than the issues related to the moral domain (Conry-Murray & Turiel, 2012).

These findings are in line with the data set forth in this research project. Thereby, the children of the sample did not choose randomly when comparing moral transgressions to the breaking of social conventions and, more interestingly, the majority of them judged the former rather than the latter more severely. This evidence is coherent with the assumption that before finishing their preschool time, a significant number of children are conscious of the fact that transgressions that affect others’ welfare or rights are more significant than conventions and social rules. A striking finding that is in keeping with this statement is the fact that age has no influence on this abovementioned tendency. Other authors have also reported the similarities appearing in moral judgments expressed by individuals from different age groups (Pellizzoni et al. 2010).

Much of this consideration is equally valid in the case of the choice that the children of the sample had to carry out to decide the most negative option between the environmental harmful conduct and the violation of social rules. Once again, the results obtained indicate that the children did not judge by chance and the majority of them pointed out that the behaviour that may cause damage to plants is more serious than the breaking of social conventions. In addition, no differences have been found among individuals belonging to different age groups. These trends are consistent with Hussar and Horvath (2011), to the extent that these authors also confirm that young children consider environmentally harmful behaviour more serious than social-conventional transgressions. Likewise, the presented data agrees with those investigations which claim that even young children consider that the environment deserves a singular moral status when it comes to evaluating the actions that human beings carry out in the natural world(Ergazaki & Andriotou, 2010; Severson & Kahn, 2010; Schmidt, 2011).

Moving on to the results concerning *the living/non-living distinction test*, it is worth noting that overall the children of the sample studied showed a significant lack of understanding of the differentiation between living beings and non-living entities. In this respect, none of the children at preschool levels (that is, those who are in the 4–5 and 5–6 age groups) and furthermore, over half of the oldest children were not able to correctly classify the four types of entities related to this study. These figures are in accordance with previous research that has made the young children’s difficulties to acquire a foundational understanding of the distinction between living beings and inanimate entities apparent (Carey, 1985; Hatano et al. 1993; Piaget, 1929; Slaughter et al. 1999).

Moreover, it is also noteworthy that the youngest children stand out among the other age groups in the sample because of their significant tendency to regard vehicles as living beings. In addition, it is also interesting to note that as a general rule, the younger the subjects of the sample are, the more frequent the trend is not to consider plants as living entities. Once again these findings are in line with previous research which indicates that young children tend to attribute animacy to a wide set of entities that includes moving objects (the sun and clouds) and, additionally, they tend to be reluctant to judge plants to be alive (Anggoro et al. 2005; Gatt et al. 2007; Leddon et al., 2009, 2011; Opfer & Siegler, 2004; Solomon & Zaitchik, 2012).

Taken as a whole, the data concerning the rate of successful classifications in each age group, it seems that age is a crucial factor in the comprehension of the animate/inanimate distinction. Accordingly, the older children of the sample demonstrated the best understanding of the concept of living beings. This is reflected in the fact that 6–7 year old individuals not only attribute the status of living being to animals and plants more often but, also, they more frequently classify entities such as vehicles, machinery, clouds and, also, the sun as inanimate. This conclusion is consistent with the claim raised by some scholar in the sense that the concept of animacy is developed progressively during childhood (Yorek et al. 2009). According to the data gathered in this research project, this process will probably involve both the progressive correct allocation of the notion of animacy to animals and plants and, also, the reconceptualization of the previous understanding of what a living being is in order to remove some inert entities from this category, initially considered alive.

In a significant study concerning the analysis of the young children’s ecological reasoning, Ergazaki and Andriotou (2010) account for the fact that children as young as 4–5 years of age can reflect on human interventions within a forest ecosystem on the basis of the intrinsic respect that flora deserves due to being living entities. More interestingly, the authors point out that this kind of judgment indicates necessary essential knowledge concerning the “living-non living” distinction in early childhood.

In connection with this idea, the data obtained in the present research by the *living/non-living distinction test* in conjunction with that provided by *the environmental judgment test* indicates a significant but also, paradoxical conclusion: age is associated with the understanding of the notion of living beings; however, it is not related to either environmental or moral judgment. In other words, a broad number of the subjects in the sample (over half of them) demonstrate a clear awareness that actions against nonhuman living beings are more serious than social-conventional transgressions but, simultaneously, only a minority of the children, belonging specifically to the oldest age group, show a suitable understanding of what a living being is.

This observation becomes more paradoxical as one considers the data provided by the analysis of the youngest children’ responses. In this respect, the 4–5 year old children show the most significant lack of understanding of what a living being is. This conclusion comes mainly from the quantitative data previously presented but it may be also interesting to point out that the researcher observed, when interviewing the children, that the youngest subjects felt confused and puzzled when attempting to answer *the living/non-living distinction test*. Despite this fact, the data also indicates that there is no difference among the three age groups concerning the belief that damaging a flower or a tree is more objectionable behaviour than *nose picking* or *regrettable table manners*.

Consequently, this study indicates that the establishment of the normative criteria that lead young children in the sample to judge environmentally harmful actions more severely than the breaking of social rules is developed prior to the full understanding of the concept of living beings. Moreover, the data reveals that, to some extent, both domains of knowledge (the normative thinking and the biological understanding) are, at least initially, unrelated.

This thought-provoking conclusion leaves open the question concerning how young children may develop the environment judgment linked to nonhuman living beings before they achieve a full understanding regarding what a living being is.

In this regard a significant explanatory framework may come from the consideration of the role that emotions, sympathy and intuition seem to play in the foundation of normative judgment (Mason, 2011 paradigm (Goodenough & Prehn, ). From this standpoint, also known as the *intuitionism*^2004^), it is believed that unconscious emotional processing is responsible for the majority of the usual moral judgments and that “most of the action in moral judgment is in the automatic, affectively laden intuitions, not in conscious verbal reasoning theory” (Haidt, 2008, p.70). Therefore, the conscious and verbally expressed evaluations on actions will play a minor role when it comes to examining an individual’s normative judgment and it is thought that *right versus wrong* evaluative feelings regarding acts or characters happen “without any conscious awareness of having gone through steps of search, weighing evidence, or inferring a conclusion” (Haidt & Bjorklund, 2008, p. 188).

Thus, despite the reflection raised in Ergazaki and Andriotou (2010) that young children’s environmental reasoning seems to indicate a rudimental understanding of the concept of living beings and, also, despite the Hussar and Horvath (2011) on the issue of the existence of a differentiated normative domain for environmental judgment in the case of young children; the fact is that, according to the data provided by the present research and in light of the aforementioned *intuitionism* paradigm, it seems more acceptable to consider that young children might produce their environmental judgment independently of any rational justifications keeping them separately from their conceptual skills linked to the biological domain.

Therefore, the choice that young children express when having to decide the most negative alternative between environmentally harmful behaviour and the socio-conventional rule breaking is immediate and spontaneous, somewhat similar to an aesthetic evaluation. Moreover, this choice will be based on previously well established emotions, sympathy and intuition towards others, including nonhuman living beings.

Looking ahead, the aforementioned conclusions suggest examining the cultural usages linked to biological information during childhood as a way to look into the social practices that lead to the adoption of the feelings and emotions that eventually paves the way for young children to develop early environmental judgment, even before being aware of what the concept of living being represents (Rigney & Callanan, 2011). In this respect, it has been pointed out that living being related topics are not only a very common matter in books for young children but also this theme is overrepresented in comparison to other science subjects such as, physical and earth science (Sackes et al. 2009; Saracho & Spodek 2010). Moreover, it is worth noting that by the age of 4 children can acquire accurate information of the biological world from picture-books and they are also capable of using this information to explain real situations concerning living beings (Ganea et al. 2011).

## References

[CR1_149] (1990). Manners.

[CR2_149] Anggoro FK, Waxman SR, Medin DL, Bara B, Barsalou L, Bucciarelli M (2005). The effects of naming practices on children’s understanding of living things. Proceedings of the twenty-seventh annual meeting of the cognitive science society.

[CR3_149] Arora L, Agarwal S (2011). Knowledge, attitude and practices regarding waste management in selected hostel students of university of Rajasthan, Jaipur. Int J Chem Environ Pharm Res.

[CR4_149] Beck JM (2006). Geopolitical imaginations of the Basque homeland. Geopolitics.

[CR5_149] Caravita S, Falchetti E (2005). Are bones alive?. J Biol Educ.

[CR6_149] Carey S (1985). Conceptual change in childhood.

[CR7_149] Cenoz J, Cenoz J, Genesse F (1998). Multilingual education in the Basque country. Beyond bilingualism: multilingualism and multilingual education.

[CR8_149] Conry-Murray C, Turiel E (2012). Jimmy’s Baby doll and Jenny’s truck: young Children’s reasoning about gender norms. Child Development.

[CR9_149] Davis J (2009). Revealing the research ‘hole’ of early childhood education for sustainability: a preliminary survey of the literature. Environ Educ Res.

[CR10_149] Davis JM (2010). Young children and the environment: early learning for sustainability.

[CR11_149] El-Hani CN (2008). Theory-based approaches to the concept of life. J Biol Educ.

[CR12_149] Ergazaki M, Andriotou E (2010). From “forest fires” and “hunting” to disturbing “habitats” and “food chains”: Do young children come up with any ecological interpretations of human interventions within a forest?. Res Sci Educ.

[CR13_149] Ganea PA, DeLoache JS, Ma L (2011). Young Children’s learning and transfer of biological information from picture books to real animals. Child Development.

[CR14_149] Gatt S, Tunnicliffe SD, Borg K, Lautier K (2007). Children’s Ideas about plants. J Biol Educ.

[CR15_149] Gomboli M (1997). Ecoeducación.

[CR16_149] Goodenough OR, Prehn K (2004). A neuroscientific approach to normative judgment in law and justice. PhilosTrans R Soc Biol Sci.

[CR17_149] González LM, Meinardi EN (2011). The role of teleological thinking in learning the Darwinian model of evolution. Evol: Education and Outreach.

[CR18_149] Haidt J (2008). Morality. Perspect Psychol Sci.

[CR19_149] Haidt J, Bjorklund F, Sinnott-Armstrong W (2008). Social intuitionists answer six questions about morality. Moral psychology.

[CR20_149] Hatano G, Siegler RS, Richards DD, Inagaki K, Stavy R, Wax N (1993). The development of biological knowledge: a multi-national study. Cogn Dev.

[CR21_149] He X, Hong T, Tiefenbacher J (2011). A comparative study of environmental knowledge, attitudes and behaviors among university students in china. Int Res Geographical Environ Educ.

[CR22_149] Hussar KM, Horvath JC (2011). Do children play fair with mother nature? understanding children’s judgments of environmentally harmful actions. J Environ Psychol.

[CR23_149] Inagaki K, Hatano G, Vosniadou S (2008). Conceptual change in naive biology. International handbook of research on conceptual change.

[CR24_149] Karniol R (2011). The color of Children’s gender stereotypes. Sex Roles.

[CR25_149] Keil FC (2010). The feasibility of folk science. Cogn Sci.

[CR26_149] Kelemen D, Rosset E (2009). The human function compunction: teleological explanation in adults. Cognition.

[CR27_149] Kelemen D, Callanan MA, Casler K, Pérez-Granados D (2005). Why things happen: teleological explanation in parent–child conversations. Dev Psychol.

[CR28_149] Kesselring T, Müller U (2011). The concept of egocentrism in the context of Piaget’s theory. New Ideas in Psychology.

[CR29_149] Knight N (2010). The role of victimization in normative judgment and justification: an empirical investigation. Philos Psychol.

[CR30_149] Kohlberg L, Goslin DA (1969). Stage and sequence: the cognitive-developmental approach to socialization. Handbook of socialization theory and research.

[CR31_149] Kohlberg L (1981). The philosophy of moral development.

[CR32_149] Kopnina H (2011). Kids and cars: environmental attitudes in children. Transport Policy.

[CR33_149] Kossack A, Bogner F (2012). How does a one-day environmental education programme support individual connectedness with nature?. J Biol Educ.

[CR34_149] Leddon EM, Waxman SR, Medin DL (2009). Unmasking “alive”: Children’s appreciation of a concept linking All living things. J Cogn Dev.

[CR35_149] Leddon EM, Waxman SR, Medin DL (2011). What does it mean to ‘live’ and ‘die’? a crosslinguistic analysis of parent–child conversations in English and Indonesian. Br J Dev Psychol.

[CR36_149] Lee DS, Kang HR (2012). The categorization of “bad animal” and its relation to animal appearances: a study of 6-year-old children’s perceptions. J Soc Evol Cultural Psychology.

[CR37_149] Lindeman M, Saher M (2007). Vitalism, purpose and superstition. Br J Psychol.

[CR38_149] Mason K (2011). Moral psychology and moral intuition: a Pox on All your houses. Australas J Philos.

[CR39_149] Margett TE, Witherington DC (2011). The nature of Preschoolers’ concept of living and artificial objects. Child Development.

[CR40_149] Molina M, Van de Walle GA, Condry K, Spelke ES (2004). The animate-inanimate distinction in infancy: Developing sensitivity to constraints on human actions. J Cogn Dev.

[CR41_149] Murphy GL, Mechelen IV, Hampton J, Michalski R, Theuns P (1993). Theories and concept formation. Categories and concepts: theoretical views and inductive data analysis.

[CR42_149] Mutisya SM, Barker M (2011). Pupils’ Environmental awareness and knowledge: a springboard for action in primary schools in Kenya’s rift valley. Sci Educ Int.

[CR43_149] Opfer JE, Siegler RS (2004). Revisiting preschoolers' living things concept: A microgenetic analysis of conceptual change in basic biology. Cogn Psychol.

[CR44_149] Osborne R, Freyberg P (1985). Learning in Science. The implications of children’s science.

[CR45_149] Onura A, Sahina E, Ceren Tekkaya C (2011). An investigation on value orientations, attitudes and concern towards the environment: the case of Turkish elementary school students. Environ Educ Res.

[CR46_149] Pellizzoni S, Siegal M, Surian L (2010). The contact principle and utilitarian moral judgments in young children. Dev Sci.

[CR47_149] Piaget J (1929). The Child’s conception of the world.

[CR48_149] Rigney JC, Callanan MA (2011). Patterns in parent–child conversations about animals at a marine science center. Cogn Dev.

[CR49_149] Rioux L (2011). Promoting pro-environmental behaviour: collection of used batteries by secondary school pupils. Environ Educ Res.

[CR50_149] Robelia B, Murphyb T (2011). What do people know about key environmental issues? a review of environmental knowledge surveys. What do people know about key environmental issues? a review of environmental knowledge surveys. Environ Educ Res.

[CR51_149] Sackes M, Trundle KC, Flevares LM (2009). Using Children’s literature to teach standard-based science concepts in early years. Early Childhood Educ J.

[CR52_149] Sanefuji W, Wada K, Yamamoto T, Shizawa M, Matsuzaki J, Mohri I, Ozono K, Taniike M (2011). One-month-old infants show visual preference for human-like feature. Lett Evol Behav Sci.

[CR53_149] Slaughter V, Jaakkola R, Carey S, Siegal M, Peterson C (1999). Constructing a coherent theory: Children’s biological understanding of life and death. Children’s Understanding of biology and health.

[CR54_149] Saracho ON, Spodek B (2010). Families’ Selection of Children’s literature books. Early Childhood Educ J.

[CR55_149] Scheinholtz L, Holden K, Kalish CW, Lamdin D (2010). Cognitive development and Children’s understanding of personal finance. Financial decisions across the lifespan: problems, programs, and prospects.

[CR56_149] Schmidt K (2011). Concepts of animal welfare in relation to positions in animal ethics. Acta Biotheoretica.

[CR57_149] Schroeder M, Graham SA, McKeough A, Stock H (2010). Gender differences in preschoolers’ understanding of the concept of life. J Early Childhood Res.

[CR58_149] Severson RL, Kahn PH (2010). In the orchard: farm worker children’s moral and environmental reasoning. J Appl Dev Psychol.

[CR59_149] Slaughter V, Lyons M (2003). Learning about life and death in early childhood. Cogn Psychol.

[CR60_149] Smetana J, Killen M, Smetana JG (2006). Social domain theory: consistencies and variations in children’s moral and social judgments. Handbook of moral development.

[CR61_149] Solomon G, Zaitchik D (2012). Folkbiology. Wiley Interdisciplinary Reviews: Cognitive Science.

[CR62_149] Spelke ES, Kinzler KD (2007). Core knowledge. Dev Sci.

[CR63_149] Thomas P, Harker H (2000). Stop picking on Me.

[CR64_149] Tsutsumi S, Ushitani T, Tomonaga M, Fujita K (2012). Infant monkeys’ concept of animacy: the role of eyes and fluffiness. Primates.

[CR65_149] Turiel E (1983). The development of social knowledge: morality and convention.

[CR66_149] Turiel E (2002). The culture of morality: social development, context, and conflict.

[CR67_149] Villarroel JD, Miñón M, Nuño T (2011). The origin of counting: a study of the early meaning of ‘one’, ‘two’ and ‘three’ among Basque- and Spanish-speaking children. Educ Stud Math.

[CR68_149] Williams JA, Podeschi C, Palmer N, Schwadel P, Meyler D (2012). The human-environment dialog in award-winning Children’s picture books. Sociol Inq.

[CR69_149] Woodward AL, Sommerville JA, Guajardo JJ, Malle B, Moses L, Baldwin D (2001). How infants make sense of intentional action. Intentions and intentionality: foundations of social cognition.

[CR70_149] Yorek N, Sahin M, Aydın H (2009). Are animals ‘more Alive’ than plants? animistic­ anthropocentric construction of life concept. Eurasia J Mathematics, Sci Technol Educ.

[CR71_149] Zaitchik D, Solomon GE (2008). Animist thinking in the elderly and in patients with Alzheimer’s disease. Cognitive Neuropsychology.

